# Diagnostic value of LncRNA SNHG16 for osteoporotic fractures and its potential regulation of fracture healing

**DOI:** 10.1186/s41065-025-00423-6

**Published:** 2025-04-07

**Authors:** Yuanming Luo, Qingfeng Zhang, Changqing Shao, Jin Li, Jiaojiao Chen, Liang Han, Xiaowei Jiang, Li Hong

**Affiliations:** 1 Department of Orthopedics, The Affiliated Hospital of Jiaxing University, Jiaxing, 314001 China; 2 Department of Spine, Beijing University of Chinese Medicine Third Affiliated Hospital, Beijing, 100029 China; 3 Department of Orthopedics, Xuzhou Central Hospital, Xuzhou, 221009 China; 4 Department of Rehabilitation, Xuzhou Central Hospital, Xuzhou, 221009 China; 5 Department of Rehabilitation, Xuzhou Rehabilitation Hospital, Xuzhou, 220005 China; 6 Department of Laboratory, Haikou Hospital of the Maternal and Child Health, No. 6, Wentan Road, Guoxing Avenue, Qiongshan District, Haikou, 570102 China

**Keywords:** SNHG16, Osteoporotic fractures, miR-432-5p, Clinical data

## Abstract

**Background:**

Osteoporotic fractures (OPF) have a serious impact on the health of patients. It is of great importance to investigate the diagnostic effect of SNH16 on OPF and the mechanism of action to promote fracture healing.

**Methods:**

132 OPF patients and 128 OP patients were included. The levels of SNHG16, Col I, RUNX2 and OCN were evaluated by RT-qPCR. The diagnostic value of SNHG16 was evaluated by ROC curve. Cell proliferation ability was assessed by CCK-8, and apoptosis rate was detected by flow cytometry. ENCORI was used to predict the binding sites of SNHG16 with downstream target genes. DLR assay demonstrated the targeting relationship between SNHG16 and miR-432-5p.

**Results:**

SNHG16 was poorly expressed in OPF patients compared with OP patients, and its expression was lower in patients with delayed healing. In addition, in the OPF, OPG level was decreased, the level of RANKL was increased, and the balance of bone resorption formation is disrupted leading to fractures. Knockdown of SNHG16 results in decreased cell proliferation and increased apoptosis, and high SNHG16 expression decreases miR-432-5p expression, thereby increasing the levels of Col I, RUNX2 and OCN.

**Conclusion:**

Increasing SNHG16 can reduce the level of miR-432-5p thereby increasing the level of osteosynthesis proteins and restoring cellular activity, thereby promoting fracture healing.

## Introduction

Osteoporosis (OP) is a metabolic bone disease caused by a variety of factors that result in a generalized decrease in bone density and destruction of the microstructure of bone tissue [1], the most common of which is postmenopausal osteoporosis caused by oestrogen deficiency [2]. Osteoporotic fractures are one of the most serious complications of OP [3], affecting around 40% of people over the age of 50, particularly women [4]. This reduces the quality of life of OPF patients and increases the economic burden. Therefore, early diagnosis and detection are crucial for OPF intervention.

LncRNAs have great potential as diagnostic markers due to their aberrant expression in a variety of diseases [5, 6]. In addition, lncRNAs also represent a promising source of biomarkers, as they play an important role in regulating cell differentiation and the cell growth cycle, among others [7]. This could provide a new theoretical basis and direction for the diagnosis and clinical targeting of OPF.

The lncRNA small nucleolar RNA host gene 16 (SNHG16), encoded by the 7571-bp region of chromosome 17q25.1 [8], has been shown to be aberrantly expressed in a number of diseases, e.g. SNHG16 is notably downregulated in MSCs derived from OP patients [9], and also regulates osteogenic differentiation of MSCs. Furthermore, SNHG16 was shown to be significantly decreased in expression in the bones of OP model mice [10]. Further, SNHG16 may play a role in disease by targeting microRNAs (miRNAs) through the ceRNA network [11, 12]. miRNAs are a class of epigenetic regulators that post-transcriptionally regulate and silence the expression of target genes, thereby modulating the interplay between osteoblasts and osteoclasts [13]. miR-432-5p has been frequently reported in cancer, and a study by Xu et al. reported that miR-432-5p was significantly upregulated in OPF patients [14]. Previously, we found a target relationship between the two through database prediction. However, the expression of SNHG16 in OPF and the potential regulatory mechanisms of it and miR-432-5p in OPF have not been reported. Therefore, we hypothesised that SNHG16 is aberrantly expressed in OPF and may be involved in the progression of OPF by regulating the level of miR-432-5p. Based on this speculation, we investigated the expression of SNHG16 and miR-432-5p in OPF and evaluated the effects of both on the physiological functions of MC3T3-E1 cells, with the aim of providing a viable diagnostic biomarker for OPF.

## Materials and methods

### Participants

132 patients with osteoporotic fracture who attended Xuzhou Central Hospital from June 2021 to December 2023 were included, and 128 patients with osteoporosis during the same period were included as a control group. Inclusion criteria: (a) all patients were postmenopausal women; (b) natural menopause time ≥ 1 year; (c) all had a first osteoporotic fracture, and the diagnostic criteria: T-value ≤ -2.5 SD, degree of reduction in accordance with the diagnostic criteria for osteoporosis, fracture occurring as soon as subjected to a low-energy external force or during daily activities. Exclusion criteria: (a) suffered from other bone metabolic diseases; (b) suffered from malignant tumors; (c) accompanied by hyperlipidemia. The OPF patients were followed for 4 months. The patients were examined at the end of 4 months and if the x-ray showed little bone scab at the fracture end, mild decalcification, and a distinct fracture line without osteosclerosis then they were categorized into delayed fracture healing group and if there was no deformity of the fracture site after two weeks of consecutive observation then they were categorized into normal healing group. There were 95 patients with normal healing and 37 patients with delayed healing.

The study was approved by the Ethics Committee of Xuzhou Central Hospital [XZXY-LK-20210523-006], and all subjects and their families signed an informed consent form.

### Cell culture and transfected

Mouse embryonic osteoblast progenitor cells (MC3T3-E1) were obtained from the Chinese Academy of Sciences (Beijing, China). They were cultured at 37 ℃ and 5% CO_2_ concentration in MEM-α medium containing nucleosides and GlutaMAX supplements (10% FBS in the medium).

Lipofectamine 2000 (Invitrogen, USA) was used for transfection. The transfected pcDNA3.1 and siRNA were purchased from GenePharma (Shanghai, China). The pcDNA3.1 empty vector, pcDNA3.1 SNHG16, siRNA empty vector and si-SNHG16 were transfected into MC3T3-E1 cells according to the manufacturer’s instructions.

### RNA extraction and Real-time quantitative reverse transcription PCR (RT-qPCR)

At the time of consultation, 5 mL of fasting peripheral venous blood was collected from patients with OP and OPF. At the end of the follow-up visit, 5 mL of fasting peripheral venous blood was again collected from patients with OPF, and the serum was collected by centrifugation for 10 min and stored in a refrigerator at -80 ℃ for use in subsequent experiments.

Total RNA was extracted from serum and cells using TRIzol reagent (Invitrogen, USA). cDNA was reverse transcribed from RNA to cDNA using a reverse transcription kit (Invitrogen, USA). cDNA was detected on a PCR system according to the instructions of SYBR Premix Ex Taq II (Takara) for SNHG16, miR-432-5p, Col I, RUNX2 and OCN expression levels. GAPDH and U6 were used as internal references and the results were normalized using the 2^−ΔΔCt^ method.

### Cell viability assay

MC3T3-E1 cells were inoculated into 96-well plates and then cultured for 24 h until the cells adhered to the wall. The 96-well plates were removed from the incubator at 0 h, 24 h, 48 h and 72 h, and 10µL CCK-8 solution was added to each well of the plate. The plates were then further incubated in the incubator for 1 h. After removal, the absorbance values were measured at 450 nm (OD450) using an enzyme marker.

### Cell apoptosis assay

MC3T3-E1 cells were inoculated in 6-well plates and cultured for 48 h. Cells were then digested using trypsin and cell lysates were collected. Cells were stained according to the instructions of Annexin V-FITC/Propidium Iodide (PI) Apoptosis Detection Kit (Beyotime, Beijing). Finally, apoptosis was detected using flow cytometry.

### DLR assay

The binding sites of SNHG16 and miR-432-5p were predicted by ENCORI website. Vectors for wild-type (WT) and mutant (MUT) of SNHG16 were constructed based on the binding sites. The vector and miR-432-5p mimic, mimic NC, inhibitor NC and miR-432-5p inhibitor were co-transfected into MC3T3-E1 cells. Finally, the luciferase activity of the vector was detected according to the DLR kit.

### Statistical analysis

All experiments were performed in triplicate. Data were expressed as mean ± deviation. Data presented as graphs were plotted using GraphPad. Data presented in tables were analyzed using SPSS. The t-test was used for between-group tests and the chi-squared test for categorical variables. Significant differences were indicated when the *p*-value was less than 0.05.

## Results

### SNHG16 expression levels and diagnostic role

**Fig. 1:**
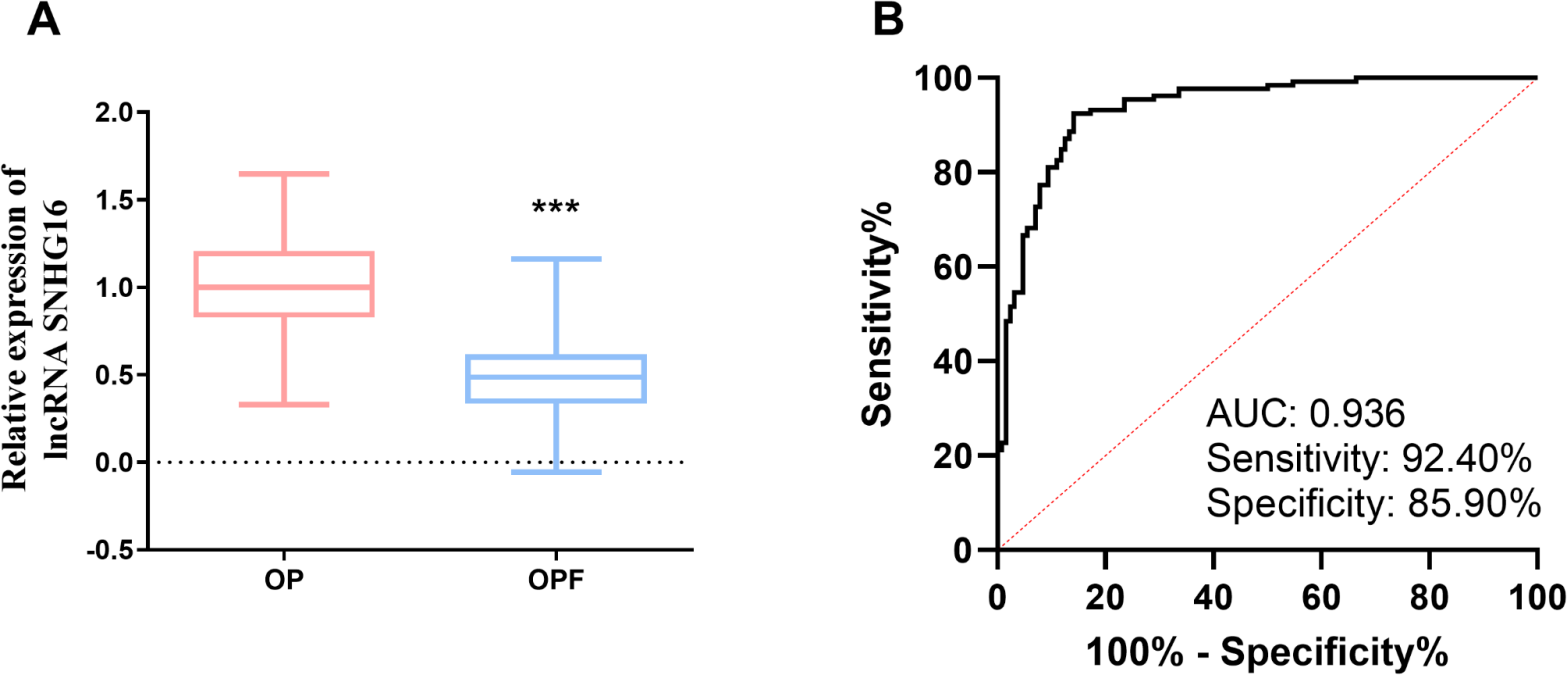
Expression of lncRNA SNHG16 in OP and OPF and its diagnostic role. **A**: Expression levels of SNHG16 in all subjects; **B**: Diagnostic value of SNHG16 in patients with osteoporotic fractures (*** *P* < 0.001 vs. OP)

Compared to the OP group, the expression level of SNHG16 was significantly decreased in the OPF group (*P* < 0.001, Fig. 1A). Furthermore, SNHG16 had a better diagnostic role with an AUC of 0.936, a specificity of 85.90% and a sensitivity of 92.40% (Fig. 1B), which allowed a better differentiation between OF and OPF patients. In addition, patients in the OP and OPF groups had significant differences in T-score, BMD, RANKL, and OPG (*P* < 0.05), whereas there were no significant differences in age, BMI, and whether or not they smoked (*P* > 0.05, Table 1).

**Table 1:** Comparison of clinical data between the OF and OPF groups

Parameter	OP Group (*n* = 128)	OPF Group (*n* = 132)	*P*
Age, years	64.24 ± 7.97	65.20 ± 7.28	0.314
BMI, kg/m^2^	24.41 ± 3.18	25.01 ± 3.19	0.136
T-score	-3.08 ± 0.41	-3.22 ± 0.43	**0.008**
Drinking			
NO	71	73	0.979
YES	57	59	
BMD, g/cm^2^	0.49 ± 0.18	0.45 ± 0.13	**0.019**
RANKL, pg/mL	266.28 ± 47.88	281.95 ± 59.12	**0.019**
OPG, ng/mL	0.77 ± 0.27	0.71 ± 0.23	**0.048**

OP, Osteoporosis; OPF, Osteoporotic fracture; BMI, Body Mass Index; BMD, Bone Mineral Density; RANKL, Receptor activator of nuclear factor kappa-B ligand; OPG, osteoprotegerin

### The correlation between SNHG16 and various indicators of OP and OPF

Indicators with significant differences between the two groups of patients were selected for Pearson analysis. The results in Table 2 indicate that SNHG16 was significantly positively correlated with T-score, BMD and OPG in the OP and OPF groups; while SNHG16 was significantly negatively correlated with the expression level of RANKL.

**Table 2:** Pearson’s analysis of the correlation between LncRNA SNHG16 and various indicators of OP group and OPF

Indicators	OP Group		OPF Group	
	*r*	*P*	*r*	*P*
T-score	0.600	< 0.0001	0.764	< 0.0001
BMD, g/cm^2^	0.707	< 0.0001	0.810	< 0.0001
RANKL, pg/mL	-0.586	< 0.0001	-0.852	< 0.0001
OPG, ng/mL	0.702	< 0.0001	0.841	< 0.0001

OP, Osteoporosis; OPF, Osteoporotic fracture; BMD, Bone Mineral Density; RANKL, Receptor activator of nuclear factor kappa-B ligand; OPG, osteoprotegerin

### SNHG16 expression in the OPF group

**Fig. 2:**
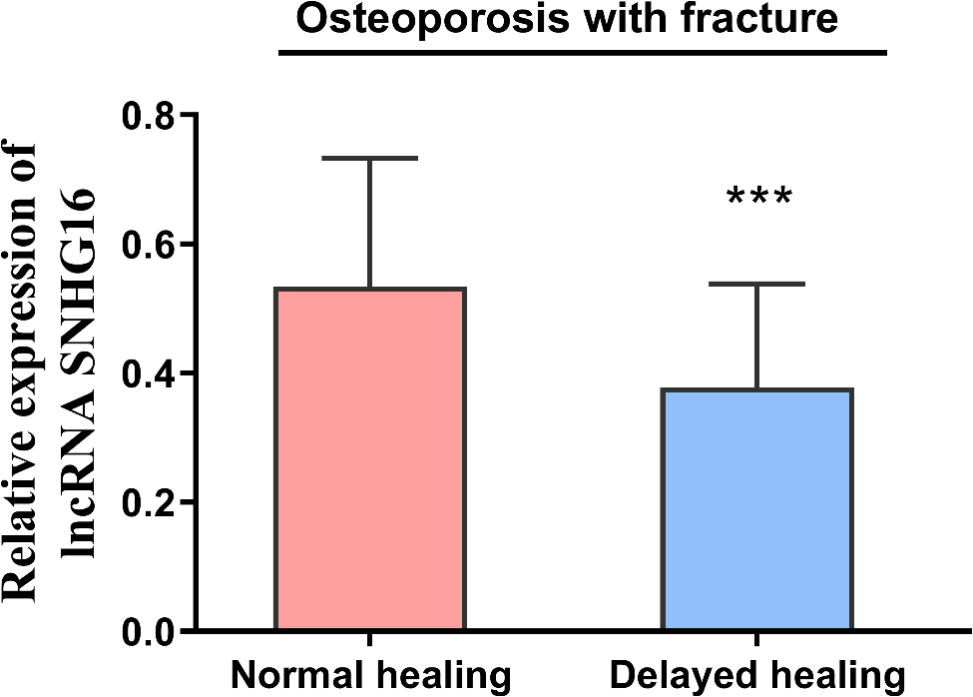
SNHG16 expression in patients with delayed and normal healing of OPF, with lower levels of SNHG16 expression in patients with delayed healing (*P* < 0.001)

SNHG16 expression levels were significantly lower in the delayed healing group than in the normal healing (*P* < 0.001, Fig. 2). In addition, there were significant differences between patients with normal healing and those with delayed healing in terms of T-score, BMD, RANKL, and OPG (*P* < 0.05), while there were no significant differences between patients with normal healing and those with delayed healing in terms of age, BMI, and whether they smoked or not (*P* > 0.05, Table 3).

**Table 3:** Comparison of indicators in patients with normal versus delayed healing in the OPF group

Indicators	Normal healing (*n* = 95)	Delayed healing (*n* = 37)	*P*
Age, years	65.23 ± 7.47	65.11 ± 6.85	0.931
BMI, kg/m^2^	24.92 ± 3.17	25.24 ± 3.29	0.609
T-score	-3.31 ± 0.41	-3.01 ± 0.41	**< 0.0001**
Drinking			
NO	57	16	0.082
YES	38	21	
BMD, g/cm^2^	0.46 ± 0.13	0.40 ± 0.13	**0.011**
RANKL, pg/mL	274.71 ± 59.98	300.54 ± 53.20	**0.024**
OPG, ng/mL	0.74 ± 0.23	0.60 ± 0.21	**0.002**

OP, Osteoporosis; OPF, Osteoporotic fracture; BMI, Body Mass Index; BMD, Bone Mineral Density; RANKL, Receptor activator of nuclear factor kappa-B ligand; OPG, osteoprotegerin

### Independent influences on delayed healing in OPF

Factors affecting delayed healing in the OPF group were analyzed by logistic binary regression. T-score (OR = 0.264), OPG (OR = 0.399) and SNHG16 (OR = 0.225) were independent factors affecting delayed healing in bone OPF. The probability of delayed healing occurring with high SNHG16 expression is 0.225 times higher than that of delayed healing occurring with low expression (Table 4).

**Table 4:** Logistic regression analyses of independent influences on delayed healing of OPF

Indicators	*P*	OR	95% CI
Age	0.821	1.105	0.464–2.634
BMI	0.793	1.215	0.285–5.184
T-score	0.024	0.264	0.083–0.836
BMD	0.066	0.403	0.152–1.064
RANKL	0.087	2.118	0.897–5.002
OPG	0.044	0.399	0.163–0.977
Drinking	0.186	2.213	0.682–7.185
LncRNA SNHG16	0.017	0.225	0.066–0.767

### Effect of SNHG16 on cells

**Fig. 3:**
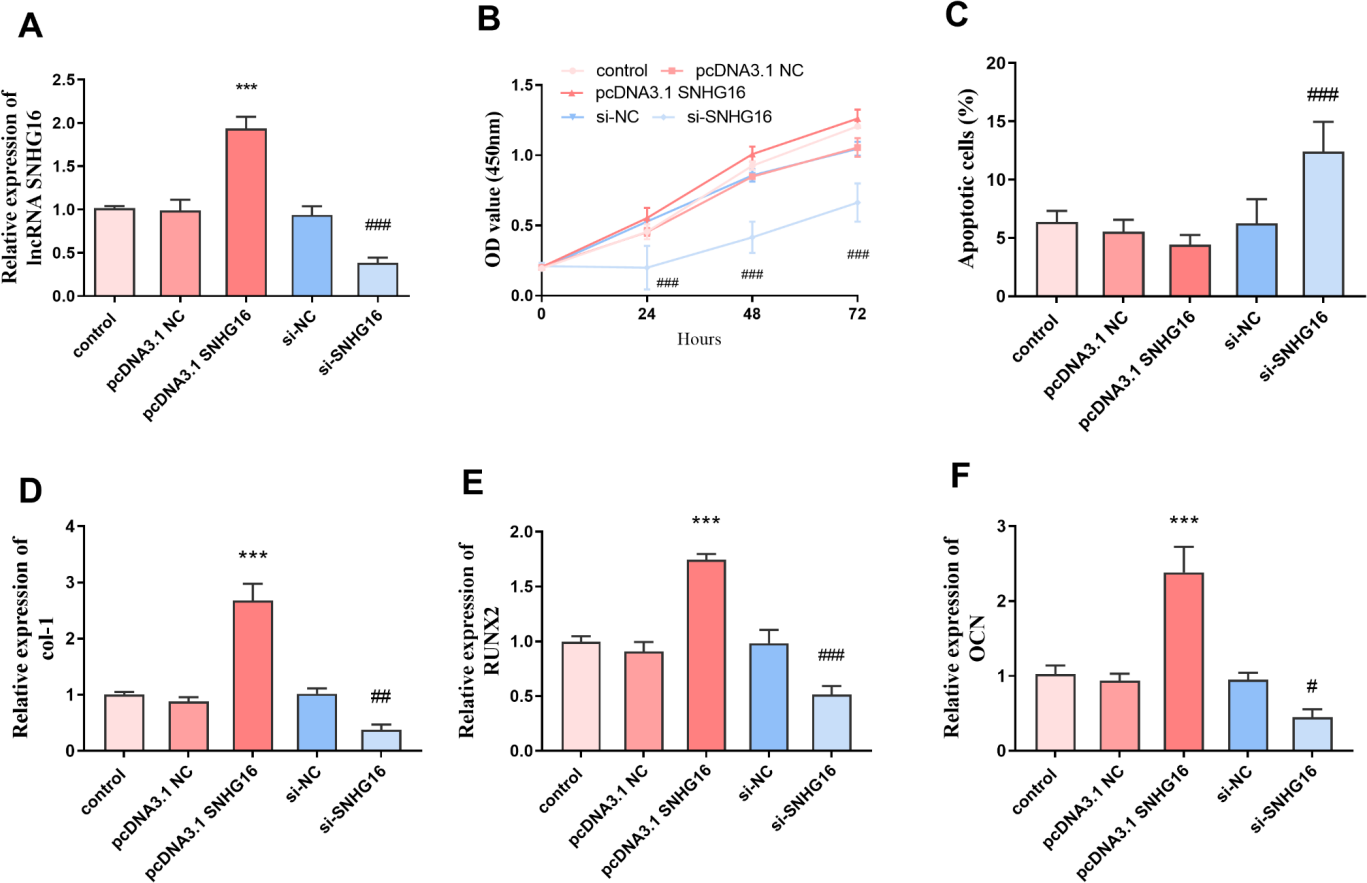
Effect of SNHG16 expression levels on cellular and bone synthesis proteins. **A**: Effects of transfection of SNHG16 expression levels; **B** and **C**: SNHG16 expression level affects cell proliferation ability and apoptosis rate; **DE** and **F**: The expression level of SNHG16 affects the expression levels of col-1, RNNX2, OCN, and all three are significantly increased with the increase in the expression level of SNHG16 (*** *P* < 0.001 vs. pcDNA3.1 NC, ### *P* < 0.001 vs. si-NC)

Transfection of SNHG16 induced a significant increase in its expression level, whereas knockdown of SNHG16 significantly downregulated its level (*P* < 0.001, Fig. 3A). After knockdown of SNHG16, the proliferation ability of cells was significantly decreased and the apoptosis rate was significantly increased; whereas, when the expression level of SNHG16 was elevated, the proliferation ability and apoptosis rate of MC3T3-E1 cells were restored to the normal level (*P* < 0.001, Fig. 3B and C). In addition, the levels of Col I, RUNX2 and OCN were notably increased after increasing the level of SNHG16, whereas the expression levels of Col I, OCN and RUNX2 were notably decreased after SNHG16 knockdown (*P* < 0.001, Fig. 3D and F).

### Downstream target genes of SNHG16

**Fig. 4:**
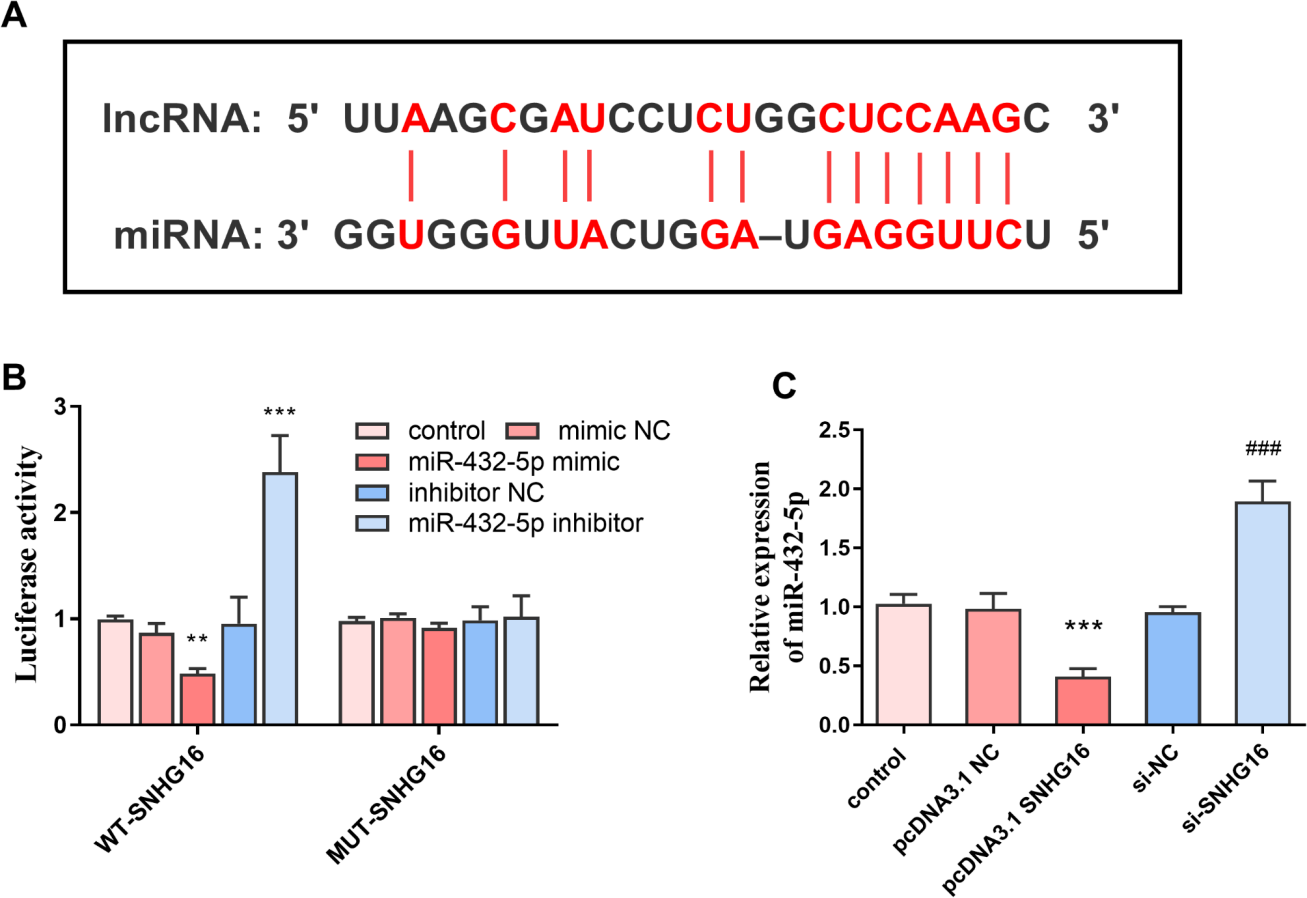
SNHG16 downstream target gene miR-432-5p. **A**: SNHG16 and miR-432-5p predicted binding sites; **B**: miR-432-5p mimic reduces luciferase activity of WT-SNHG16, whereas miR-432-5p inhibitor leads to increased luciferase activity; **C**: Transfection of SNHG16 resulted in decreased levels of miR-432-5p, while knockdown of SNHG16 resulted in upregulation of miR-432-5p levels (*** *P* < 0.001 vs. control, ### *P* < 0.001 vs. si-NC)

Figure 4A demonstrates the predicted binding sites for SNHG16 and miR-432-5p. DLR experiments demonstrated a target-binding relationship between SNHG16 and miR-432-5p. miR-432-5p mimics significantly decreased the luciferase activity of WT SNHG16, whereas the inhibitor had the opposite effect and significantly increased luciferase activity (*P* < 0.001, Fig. 4B). In addition, transfection of SNHG16 resulted in a consistent decrease in miR-432-5p levels, whereas knockdown of SNHG16 expression resulted in a significant upregulation of miR-432-5p (*P* < 0.001, Fig. 4C).

### SNHG16 co-regulates cell physiological functions with miR-432-5p

**Fig. 5:**
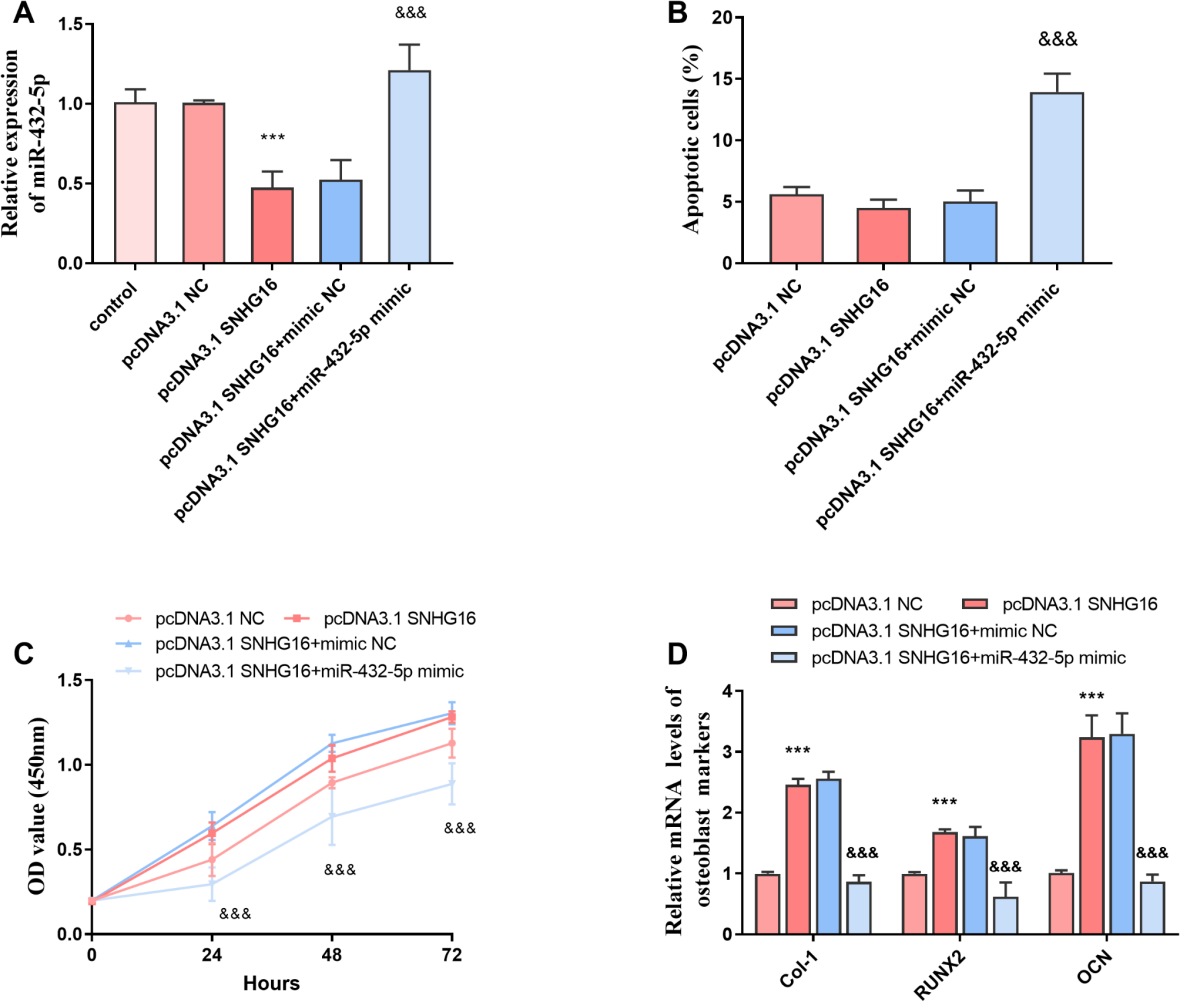
SNHG16 co-regulates cell physiological functions with miR-432-5p. **A**: Transfection of SNHG16 leads to a significant decrease in the level of miR-432-5p, while its mimics increase its expression level; **B**: Increasing the level of SNHG16 had no significant effect on the apoptosis rate, but increasing the level of miR-432-5p resulted in a significant increase in the apoptosis rate; **C**: The cell proliferation capacity after increasing the level of SNHG16 was increased but not significantly. Increasing the level of miR-432-5p resulted in a significant decrease in the proliferative capacity of the cells; **D**: After increasing the level of SNHG16, the level of miR-432-5p was decreased and the level of bone-related synthetic proteins (Col-1, RUNX2, OCN) was significantly increased. However, after increasing the level of miR-432-5p, the expression levels of Col-1, RUNX2, OCN were significantly decreased (*** *P* < 0.001 vs. pcDNA3.1 NC, &&& *P* < 0.001 vs. pcDNA3.1 SNHG16 + mimic NC)

As shown in Fig. 5A, transfection of SNHG16 resulted in a significant decrease in the level of miR-432-5p, whereas its analogue increased its expression level (*P* < 0.001). Whereas changes in SNHG16 levels had no significant effect on the apoptosis rate and proliferation capacity (*P* > 0.05), increasing the level of miR-432-5p resulted in a significant increase in the apoptosis rate and a significant decrease in the proliferation capacity of the cells (Fig. 5B-C, *P* < 0.001), which suggests that miR-432-5p may be an unfavorable factor for cell proliferation and differentiation. In addition, increasing the level of SNHG16 decreased the level of miR-432-5p and significantly increased the level of bone-associated synthetic proteins (Col-1, RUNX2, OCN). However, the expression levels of Col-1, RUNX2, and OCN were significantly decreased after increasing the levels of miR-432-5p (Fig. 5D, *P* < 0.001). This suggests that SNHG16 may have an important role in the process of bone formation, and the elevated expression level of SNHG16 can promote the healing process of OPF.

## Discussion

OP is a common chronic disease in the elderly population, especially in postmenopausal middle-aged and elderly women. For postmenopausal women, the decline of oestrogen is the main influencing factor for the occurrence of OP [15]. Moreover, with the aging of the population, the incidence of OPF is increasing year by year, which poses a serious threat to women’s health. The healing rate of OPF also affects the patients’ normal activities and heart state [16]. Therefore, it is of great significance to investigate the diagnostic role of lncRNAs in OPF and their regulation of fracture healing.

SNHG16 was first identified in neuroblastoma and was later found to be aberrantly expressed in a variety of cancers [17]. The study by Amany et al. reported that SNHG16 was aberrantly expressed in OP [10]. Our study reported for the first time that SNHG16 was lowly expressed in OPF. Aberrantly expressed lncRNAs have potential as diagnostic markers. Further results showed that SNHG16 had a good diagnostic effect on OP and OPF, with a specificity that could reach 85.90% and a sensitivity of 92.40%. The BMD of OPF patients was significantly lower than that of OP patients. In addition, nuclear factor κB receptor activator ligand (RANKL) and osteoprotegerin (OPG) are also important factors in fracture occurrence [18–20]. RANKL secreted by osteoblasts can bind to RANK on the surface of osteoclasts to activate osteoclasts and promote osteoclast differentiation and maturation [21]. While OPG is an inducible receptor for RANKL and reduces osteoclast production by binding to RANKL [22]. In premenopausal women, OPG and RANKL are in balance. In postmenopausal women, RANKL increases due to the decrease in oestrogen levels [23], and compared to the OP group, OPF patients have higher levels of RANKL and notably lower levels of OPG. Overexpression of RANKL and overactivation of osteoclasts leads to increased bone resorption, greater bone resorption than bone formation, an imbalance in bone remodeling metabolism, and decreased bone density and strength, which in turn leads to a number of bone diseases [24].

Fracture healing can be divided into 3 overlapping phases: inflammation, scab formation and bone remodeling [25]. An in-depth study of fracture healing at the cellular level may provide better diagnostic and therapeutic tools for delayed fracture healing. lncRNAs can regulate many physiological activities of osteoblasts, including cell migration, differentiation, proliferation and apoptosis [26]. Our study showed that SNHG16 is a favorable factor for fracture healing. Knockdown of SNHG16 resulted in a significant decrease in cell proliferation and a significant increase in apoptosis. In contrast, the proliferative capacity and apoptotic rate of MC3T3-E1 cells returned to normal at higher SNHG16 levels. Whereas miR-432-5p exists as an unfavorable factor in fracture healing, increasing the level of miR-432-5p resulted in a significant decrease in the proliferation ability and a significant increase in the apoptosis rate of MC3T3-E1 cells.

Collagen type I (Col I) is a major component of the extracellular matrix, which is essential for maintaining the structural integrity and strength of tissues and is abundant in connective tissues such as skin and bone [27, 28]. When SNHG16 levels were elevated, Col I levels were notably increased, whereas silencing SNHG16 expression and increasing miR-432-5p expression levels both led to a decrease in Col I levels. OCN is often used as a serum marker of osteoblast bone formation [29]. Runt-related transcription factor 2 (Runx2) is a key regulator of osteoblast differentiation [30]. Our results show that high levels of SNHG16 increase the expression levels of OCN and Runx2. Runx2 also promotes fracture healing by facilitating OPG binding to RANKL and reducing osteoclast production [31]. In addition, we looked for the downstream target gene miR-432-5p of SNHG16.

However, our current study has some limitations: firstly, the current findings remain to be validated in a larger independent patient cohort. Therefore, in future studies, we will include more patients to validate the current experimental results and improve the sampling time to compare the expression of target genes in fasting and non-fasting blood samples; second, the signaling pathway of SNHG16 targeting miR-432-5p to regulate the involvement of MC3T3-E1 cells in fracture healing remains to be further investigated. In the subsequent experiments, we will consider exploring the signaling pathway of SNHG16 targeting miR-432-5p involved in fracture healing through GO and KEGG databases and validate it by experiments.

In conclusion, the expression level of SNHG16 is decreased in patients with OPF. SNHG16 has great potential to become a diagnostic marker for OPF. Increasing the expression of SNHG16 can reduce the level of miR-432-5p, promote cell proliferation, improve cell activity, reduce apoptosis, and increase the content of osteosynthesis proteins, which may be involved in promoting fracture healing.

## Data Availability

The datasets used and/or analyzed during the current study are available from the corresponding author on reasonable request.
